# Impact of Lung Function Decline on Mortality in Lung Transplant Recipients: Long-Term Results From the L-CsA-i Study for the Prevention of Bronchiolitis Obliterans Syndrome

**DOI:** 10.3389/fmed.2022.897581

**Published:** 2022-06-02

**Authors:** Nikolaus Kneidinger, Alessandro Ghiani, Katrin Milger, Víctor Monforte, Christiane Knoop, Peter Jaksch, Jasvir Parmar, Piedad Ussetti, Amparo Solé, Joachim Müller-Quernheim, Andreas Voelp, Juergen Behr, Claus Neurohr

**Affiliations:** ^1^Department of Medicine V, University Hospital, LMU Munich, Comprehensive Pneumology Center (CPC-M), Member of the German Center for Lung Research (DZL), Munich, Germany; ^2^Department of Pulmonology and Respiratory Medicine, Robert-Bosch-Hospital, Stuttgart, Germany; ^3^Hospital Universitari Vall d'Hebron, Barcelona, Spain; ^4^CHU Erasme Université Libre de Bruxelles, Brussel, Belgium; ^5^Department of Thoracic Surgery, Medical University of Vienna, Vienna, Austria; ^6^Royal Papworth Hospital, Cambridge, United Kingdom; ^7^Hospital Universitario Puerta de Hierro, Madrid, Spain; ^8^Hospital Universitario La Fe, Valencia, Spain; ^9^Department of Pneumology, Medical Center - University of Freiburg, Faculty of Medicine, University of Freiburg, Freiburg im Breisgau, Germany; ^10^Consultant, Hamburg, Germany

**Keywords:** lung transplantation, CLAD, chronic rejection, BOS, Cyclosporine (CsA)

## Abstract

**Background:**

Chronic lung allograft dysfunction (CLAD) is defined by a progressive loss of FEV1 and is associated with premature mortality. The aim of this study was to investigate the direct association between FEV1 decline and risk of mortality in patients after lung transplantation (LTx).

**Methods:**

10-year follow up data from lung transplant recipients participating in randomized placebo-controlled clinical trial investigating the role of liposomal Cyclosporine A for inhalation (L-CsA-i) in the prevention of bronchiolitis obliterans syndrome (NCT01334892) was used. The association between the course of FEV1 over time and the risk of mortality was assessed using joint modeling and Cox regression analysis.

**Results:**

A total of 130 patients were included. Predictors of FEV1 decline were a higher absolute FEV1 at baseline and male sex. The joint model analysis indicated a significant association of change of FEV1 and risk of mortality (*p* < 0.001), with a predicted 3.4% increase in mortality risk for each 1% decline in FEV1. Significant predictors of a progressive phenotype were single LTx and treatment with placebo (as opposed to L-CsA-i). At the end of follow-up, 82 patients (63.1%) were still alive. Cox regression analyses for mortality identified only single LTx as a predictor of higher risk.

**Conclusion:**

Based on our observation of a close association between FEV1 and mortality over a period of 10 years we suggest FEV1 as a valid predictor of mortality and a suitable surrogate endpoint in the investigation of early interventions.

## Introduction

Chronic lung allograft dysfunction (CLAD) has been proposed as an umbrella term to describe the clinical manifestations of a range of pathological processes in the airway and parenchymal compartments of the lung allograft that lead to a significant and persistent deterioration in lung function (1). Clinical trials investigating prophylaxis and therapy of CLAD to improve outcome are based on the assumptions that a decline in the forced expiratory volume in 1 s (FEV1) is a surrogate of mortality. However, even though there is common consensus that progressive loss of lung function leads to earlier death in this population, empirical data that support this assumption are sparse.

In 2009, PARI Pharma GmbH (Gräfelfing, Germany) initiated a randomized, placebo-controlled multicenter trial (Study No. 12011.201) in 130 patients who had undergone lung transplantation (LTx), with the aim of preventing or delaying the onset of bronchiolitis obliterans syndrome (BOS) by means of inhalation of liposomal Cyclosporine A (L-CsA-i) (ClinicalTrials.gov, Identifier: NCT01334892). Patients were eligible to participate in the trial 6–32 weeks after transplantation and were randomized to either L-CsA-i or placebo. The sponsor decided to terminate the study early due to an unexpectedly low incidence of BOS, which would have required an extension of the study period by an estimated 3.5 years at least. Although the study failed to meet its primary endpoint, the results were encouraging (2). A non-significant 2-year actuarial treatment difference in BOS-free survival of 14.1% in favor of L-CsA-i was observed in the full analysis population.

The study has demonstrated that a preventive trial with the endpoint CLAD is difficult to realize. Long observation time and a high number of patients would be necessary for a statistically sufficient number of events. Therefore, the use of FEV1 as a surrogate of survival would facilitate trial design. However, the association and its magnitude of FEV1 and survival in CLAD-free individuals early after transplantation remains poorly understood.

Therefore, from the former participants of the above mentioned BOS prevention trial, approximately semiannual 10-year follow-up data for FEV1 and patient survival have now been acquired in accordance with local routine practice.

The aim of the study was to investigate the association between FEV1 decline over time and mortality in patients with single (SLTX) and double LTx (DLTX) and with L-CsA-i or placebo treatment received during PARI study no. 12011.201. Furthermore, we aimed to assess factors associated with a progressive phenotype and associated mortality.

## Methods

### Study Design and Participants

Data were acquired during a non-interventional, retrospective follow-up investigation that included randomized participants of PARI Study No. 12011.201. In the period in which patient were actively participating in the original trial, FEV1 measurements were performed every 2 months. For the retrospective follow-up period FEV1 measurements from every half-year were requested for up to 10 years from the participating centers. Information was taken from the patients' medical records. No study specific visits or investigations were performed. The study was approved by the central institutional ethics committee (Munich, Germany; project number 20-701).

All randomized subjects of PARI Study No. 12011.201 were eligible for participation in the retrospective follow-up. For eligibility for inclusion into Study No. 12011.201, subjects had to be recipients of a SLTX or DLTX between 6 and 32 weeks before the start of randomized treatment and, had to have a life expectancy of at least 6 months, and had to be free of CLAD as described previously (2). Patients were randomized to one of the two treatments, L-CsA or placebo which was administered twice daily using PARI Pharma's Investigational eFlow® nebulizer system.

### Outcomes

Baseline data included type of lung transplantation (SLTX vs. DLTX), randomized treatment (L-CsA-i or placebo), date of LTx, date of randomization. Follow-up data for each time point included date and absolute FEV1, subject survival at end of follow-up, and date of death (if applicable).

The baseline used during the analyses was the individual date of randomization into PARI Study No. 12011.201. FEV1 was expressed in % of the individual FEV1 value (FEV1_baseline_%) obtained at the baseline visit of the original randomized trial. The reference value for determining FEV1 percent decline was the single FEV1 baseline value of PARI Study No. 12011.201, not the personal best value after LTx. FEV1_baseline_% of the baseline value was calculated as:


$$\begin{array}{l} \text{Current FEV}1\;\left(\text{L}\right)/\text{FEV}1\text{ value at baseline }\left(\text{L}\right)\times \;100. \end{array}$$

Identified causes of death were classified by an expert panel as CLAD-related or non-CLAD-related. Death from an unknown cause was to be considered CLAD-related. In subjects still alive at the end of the follow-up period, the date of the last contact alive was assumed to be the date of the last documented FEV1 assessment.

### Statistics

The association between post-transplant FEV1 decline over time and mortality was analyzed using joint modeling (3). A joint model was fitted using FEV1 serial measurements over time as a covariate and time between baseline and death as a time-to-event endpoint. For the serial measurements part of the analysis, a linear mixed model (LMM) was used. The timing of the measurements of FEV1 was analyzed according to the actual documented date of the examinations. Serial measurements modeling was performed using all available data, i.e., starting at baseline of the PARI trial and ending at the individual end of follow-up. Type of lung transplantation (SLTX or DLTX), absolute FEV1 at baseline, age, sex, and linear as well as quadric time by treatment interaction (L-CsA-i or placebo) were included as additional fixed factors or covariates. For the time-to-event process treatment, type of LTx, absolute FEV1 at baseline, age, and sex were used as additional covariates. Time between baseline and persistent decline in measured FEV1 value from the reference (i.e., FEV1_baseline_% ≥ 20%) were modeled using multiple Cox regression analysis. The following variables were defined as covariates: treatment, type of LTx, sex, age at baseline, and FEV1 (absolute) at baseline.

Time between baseline and death was modeled using multiple Cox regression analysis. The following variables were defined as covariates: treatment (L-CsA-i or placebo), type of LTx, sex, age at baseline, and FEV1 (absolute) at baseline. Treatment and type of LTx were always retained in the model (forced entry). All other covariates were selected using backward removal.

The joint model assessing the association between FEV1_baseline_% and mortality risk was computed using function jointModel of the R package JM version 1.4–8 under R version 4.0.3 (4). All other statistical computations were performed in IBM SPSS version 24.

## Results

### Participant Characteristics

For the follow-up study, complete data sets could be retrieved for all randomized participants of centers Vienna, Brussels, Munich, Freiburg, Cambridge, Madrid, Barcelona, and Valencia of PARI study no. 12011.201, together representing 118 (90.8%) out of a total of 130 subjects randomized into the trial. Mean follow-up was 61.4 ± 38.0 months. For the remaining 12 subjects, only the data documented during PARI Study No. 12011.201 were included into the analysis, and the subjects were censored at the last documented study visit. One of the participating sites reported only date of death (if applicable), and FEV1 and date for last contact alive but no intermediate FEV1 follow-up assessments.

In total, 67 of the 130 subjects (51.5%) had chronic obstructive pulmonary disease (COPD) as underlying diagnosis, 37 (28.5%) had interstitial lung disease (ILD), 13 (10.0%) had cystic fibrosis (CF), and the remaining 13 (10%) presented with other underlying diagnoses. In SLTX (*n* = 40) the proportion of subjects with COPD was 35% compared to 60% with ILD. In DLTX (*n* = 90), 51.5% had COPD, 14.4% had ILD and 14.4% had CF. Basic participant characteristics are summarized in Table 1. A detailed characterization of the study population has been published previously (2).

**Table 1:** Baseline characteristics of study participants.

		**L-CsA-i (*n* = 74)**	**Placebo (*n* = 56)**	**Total (*n* = 130)**
Sex: number (%) female		29 (39.2%)	25 (44.6%)	54 (41.5%)
Age at screening (years)		51.4 ± 12.8	52.1 ± 10.1	51.7 ± 11.7
		Range 20–68	Range 24–67	Range 20–68
Type of lung transplantation	Single	23 (31.1%)	17 (30.4%)	40 (38.8%)
	Double	51 (68.9%)	39 (69.6%)	90 (69.2%)
Time between lung transplantation and baseline (weeks)		15.0 ± 7.8	18.5 ± 6.3	16.5 ± 7.4
		Range 1.6–28.9	Range 2.4–28.4	Range 1.6–28.9
FEV1 at baseline (L)		2.32 ± 0.80	2.44 ± 0.70	2.37 ± 0.76
		Range 0.96–4.93	Range 1.12–4.17	Range 0.96–4.93
Maximum FEV_1_ after lung transplantation (personal best, L)		2.67 ± 0.84	2.73 ± 0.81	2.70 ± 0.82
		Range 0.96–5.08	Range 1.21–4.50	Range 0.96–5.08

*Data are presented as mean ± SD and range or number and %*.

*FEV1, forced expiratory volume in 1 s; L-CsA-i, liposomal Cyclosporine A for inhalation*.

### Association Between Post-transplant FEV1 Decline Over Time and Mortality

**Figure 1:**
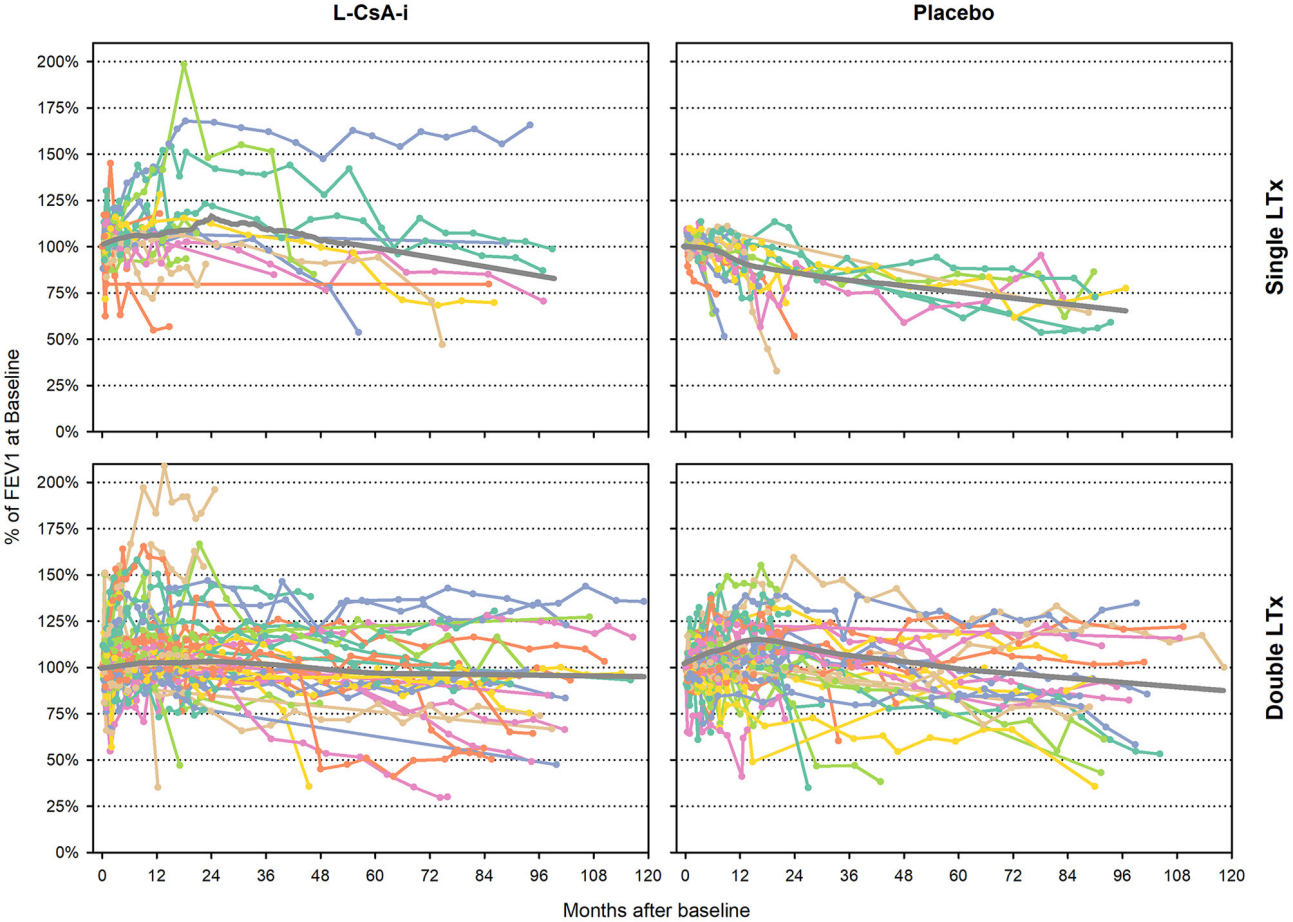
Individual subject trajectories of FEV1_baseline_% from baseline to end of follow-up. FEV1_baseline_% of intraindividual baseline value—individual subject trajectories (Month 0 corresponds to the baseline assessment of study 12011.201). Gray lines represent LOWESS regression lines.

The individual subject trajectories for FEV1_baseline_% (Figure 1) indicate a great diversity of idiosyncratic time courses. While some subjects remained on a stable level or improved after baseline, others showed a gradual deterioration of lung function or were characterized by rapid decline of lung function.

The main results of the LMM used to estimate the association of baseline variables on the longitudinal course of FEV1_baseline_% (dependent variable) as a function of time are summarized in Table 2. Table 2 indicates a strong association between FEV1_baseline_% and time, with a positive linear trend and a negative quadric (i.e., negative U-shaped) component. This may reflect the fact that subjects' lung function still tended to improve during the earlier stages of the follow-up whereas FEV1 predominantly tended to decline during later stages. Higher absolute FEV1 at baseline was associated with more pronounced post-baseline decline, with a predicted loss of 6.2% of the baseline value per liter of FEV1 at baseline. Moreover, compared to female subjects, males were predicted to have an average of 6% of additional post-baseline FEV1 compared to the baseline value.

**Table 2:** Association between FEV1 % of baseline value time course and various predictors—linear mixed model main results.

**Parameter**	**Estimate (B)**	**95% confidence interval**	***P***
Treatment: placebo	−1.431	−5.179, 2.318	0.454
Time, linear association (months)	0.149	0.007, 0.290	0.039
Time, quadric association (months^2^)	−0.005	−0.006, −0.004	<0.001
Treatment (placebo) by time interaction, linear (months)	−0.420	−0.660, −0.180	0.001
Treatment (placebo) by time interaction, quadric (months^2^)	0.002	0.000, 0.003	0.009
FEV1 at baseline (L)	−6.222	−9.333, −3.112	<0.001
Type of LTx: single	−4.714	−9.442, 0.124	0.056
Age at baseline (years)	−0.079	−0.236, 0.079	0.326
Sex: male	5.959	1.679, 10.239	0.006

*The model represents the longitudinal process of the joint model assessing the association between FEV1 % of baseline time course and patient mortality*.

*FEV1, forced expiratory volume in 1 s; LTx, lung transplantation*.

**Figure 2:**
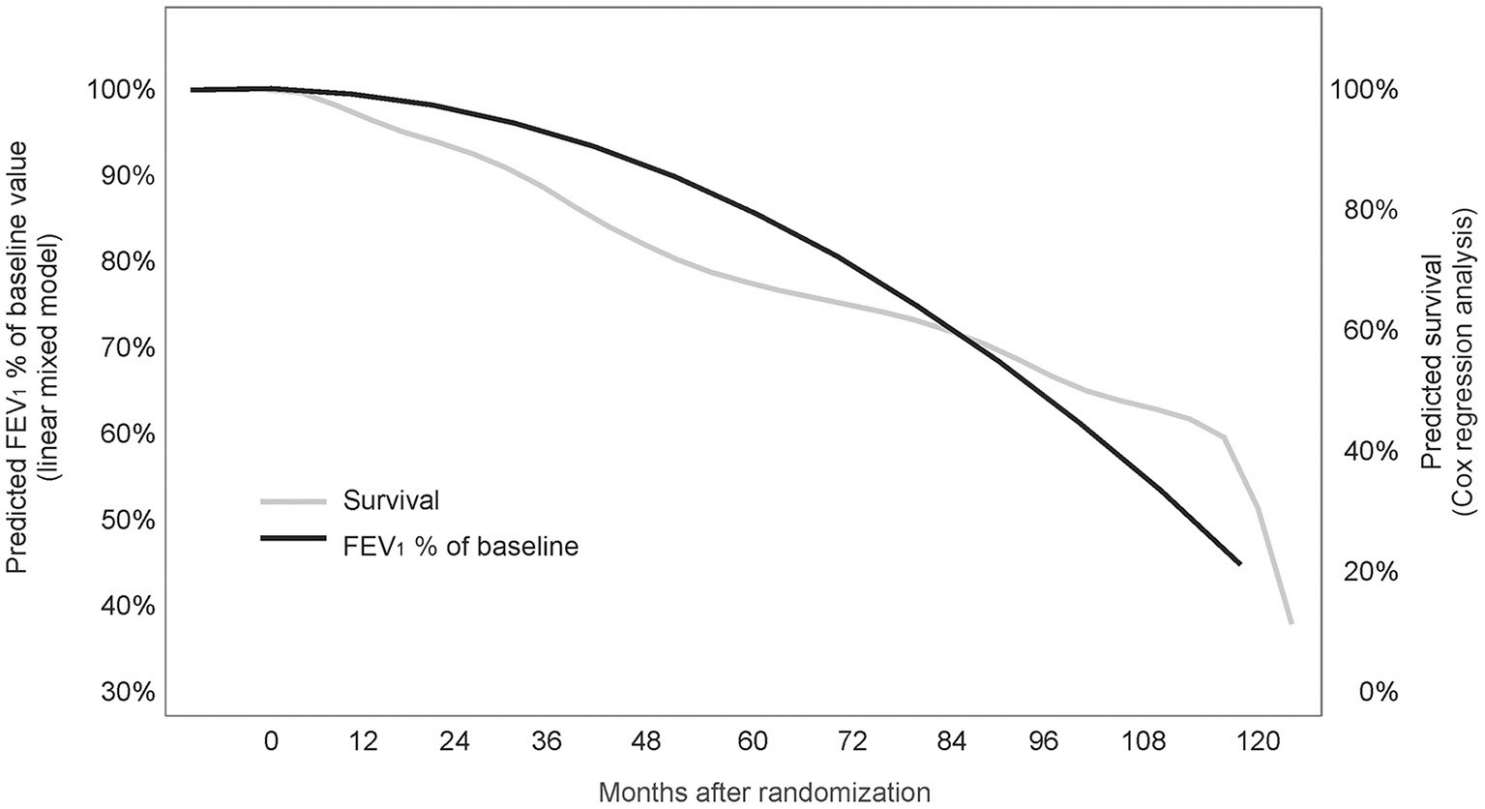
Association between FEV1_baseline_% of the baseline value and survival (joint model analysis). The predicted association between FEV1% of baseline value and mortality risk is illustrated graphically.

Using time between baseline and death as a time-to-event endpoint a highly significant association of FEV1_baseline_% with mortality risk was found [Exp(B) 0.968; 95%CI 0.952–0.984; *p* < 0.001]. According to the estimated coefficient, individuals with a 1% lower FEV1_baseline_% are at a 1/0.968 = 1.034-fold mortality risk (corresponding to 3.4% higher mortality risk) compared to an individual with a 1% higher FEV1_baseline_% preserved. Accordingly, when the difference between any two individuals in FEV1_baseline_% of maximum is 10%, the predicted mortality risk of the individual with the lower value for FEV1_baseline_% of maximum is increased by 1.034^10^ = 1.397, corresponding to a predicted risk increase by 39.7%. The predicted association between FEV1_baseline_% and mortality risk is illustrated graphically in Figure 2. Treatment (L-CsA-i or placebo) and age, which were also included into the event model as covariates, did not show a statistically meaningful predictive effect for the association between FEV1_baseline_% and mortality risk.

### Time to Progression to Allograft Dysfunction

**Figure 3:**
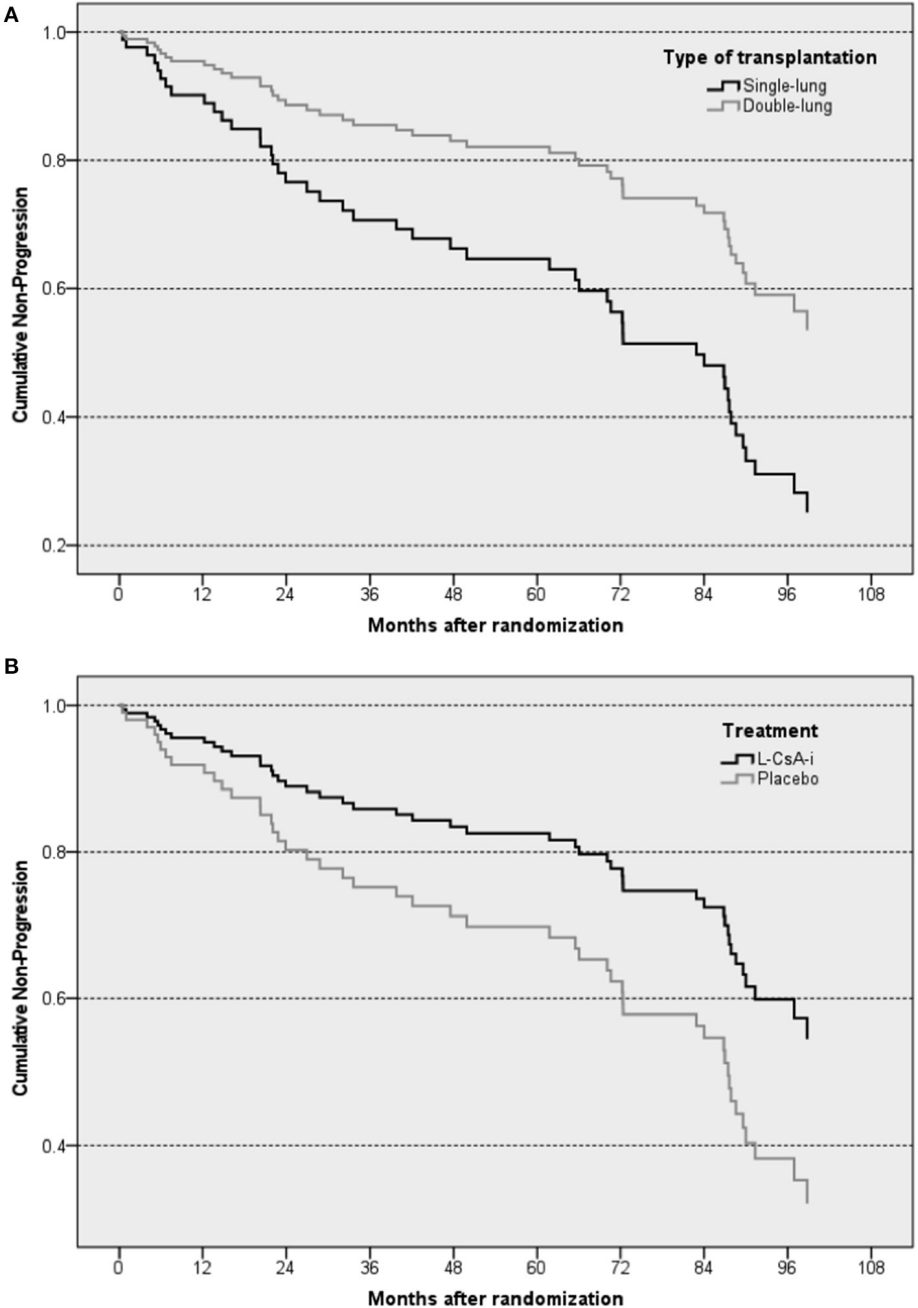
Graphical illustration of estimated cumulative non-progression to a loss in FEV1 by ≥20% of the baseline value—Cox regression analysis plotted for type of transplantation **(A)** and treatment **(B)**. The “steps” in the curves of the subsets represent predicted events resulting from the regression model.

Out of a total of 130 subjects assessed, 47 (36.2%—L-CsA 20/74, 27.0%; placebo 27/56, 48.2%) progressed to FEV1_baseline_% < 80% over the observation period. The main results of the Cox regression model predicting progression to FEV1_baseline_% < 80% are shown in Table 3. The predictive effect of the final model that included only treatment (L-CsA-i or placebo) and type of LTx as covariates was significant (−2 log likelihood: 384.7; *p* = 0.003). Even though both covariates were included into the model using forced entry, both were also found to have a significant predictive effect. The risk of lung function progression to FEV1_baseline_% < 80% for a subject receiving L-CsA-i relative to a subject receiving placebo was estimated to be 0.533 (Table 3), and hence patients receiving L-CsA-i are predicted to be an 87.6% (1/0.533 = 1.876) lower relative risk of progression. For type of LTx, the relative risk of a subject with DLTX as compared to SLTX was estimated as 0.452, i.e., the relative risk of progression of a single lung transplant patient is predicted to be increased by factor 2.2 (1/0.452 = 2.212). Figure 3 shows the estimated curve for cumulative non-progression to FEV1 < 80% plotted for subjects with SLTX or DLTX and for those treated with L-CsA-i or placebo, respectively.

**Table 3:** Cox regression model with progression to FEV1_baseline_% ≥ 20% or below as the dependent variable and parameter estimates for covariates in the equation.

		**B**	**SE**	**Wald**	**Df**	**Sig**.	**Exp (B)**	**95.0% CI for Exp (B)**	
								**Lower**	**Upper**
First step	Treatment: L-CsA-i	−0.661	0.304	4.715	1	0.030	0.517	0.285	0.938
	Type of transplantation: double LTx	−0.787	0.351	5.021	1	0.025	0.455	0.229	0.906
	Sex: female	−0.303	0.361	0.703	1	0.402	0.739	0.364	1.499
	Age at baseline (years)	−0.009	0.014	0.395	1	0.530	0.991	0.964	1.019
	FEV_1_ at randomization (L)	−0.083	0.272	0.092	1	0.761	0.921	0.541	1.568
Last step	Treatment: L-CsA-i	−0.629	0.296	4.504	1	0.034	0.533	0.298	0.953
	Type of transplantation: double LTx	−0.795	0.302	6.932	1	0.008	0.452	0.250	0.816

*While the upper part of the table shows the initial step that includes all specified covariates, the lower part shows the final step after removal of the covariates that did not have a statistically importance, predictive effect for lung function progression*.

*FEV1, forced expiratory volume in 1 s; LTx, lung transplantation; L-CsA-i, liposomal Cyclosporine A for inhalation*.

An additional Cox regression model with time to progression to allograft dysfunction (FEV1_baseline_% < 80%) as the dependent variable that also included underlying diagnosis as a predictor was calculated *post-hoc*. The main effect of underlying diagnosis was statistically significant (*p* = 0.026). Compared to the mean risk of progression for all underlying diagnoses (deviation contrasts), COPD and CF decreased the risk while ILD and other underlying diagnoses increased the risk. No significant interaction between underlying diagnosis and type of transplantation was observed.

### Survival

**Figure 4:**
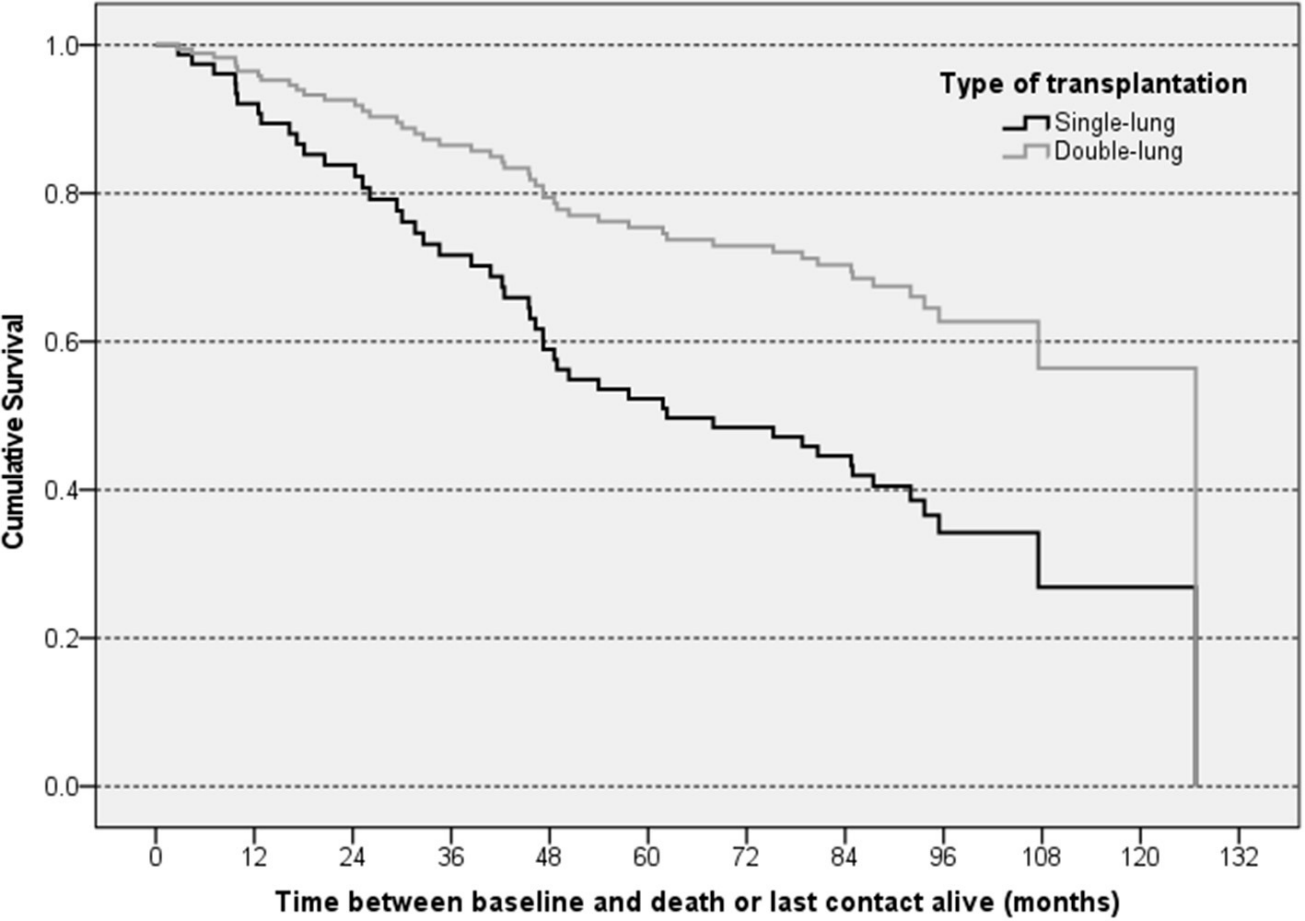
Graphical illustration of estimated cumulative survival for subjects with different types of LTx—Cox regression analysis. The “steps” in the curves of the subsets represent predicted events resulting from the regression model.

In total, 82 patients (63.1%) were confirmed to be still alive at the end of the 10-year follow-up. A total of 12 patients (25%) died during the first 2 years, and a cumulative total of 29 patients (60%) died until the end of the fourth year after baseline. The overall mortality rates were comparable for L-CsA-i (*n* = 28, 37.8%) and placebo (*n* = 20, 35.7%). Crude median survival time was 71.6 months in the L-CsA-i group and 81.0 months in the placebo group calculated from baseline of trial 12011.201. In Cox regression analysis predicting mortality, type of LTx was identified as the only covariate that had a statistically meaningful effect (Table 4). For type of LTx, the relative risk of a subject receiving a DLTX as compared to SLTX was estimated as 0.436, i.e., the relative risk of progression of a SLTX recipient is predicted to be increased by factor 2.3 (1/0.436). Figure 4 shows the estimated cumulative survival curve for subjects SLTX or DLTX.

**Table 4:** Cox regression model with all-cause mortality as the dependent variable and parameter estimates for covariates in the final equation.

		**B**	**SE**	**Wald**	**Df**	**Sig**.	**Exp (B)**	**95,0% CI for Exp (B)**	
								**Lower**	**Upper**
First step	Treatment: L-CsA-i	−0.055	0.304	0.032	1	0.857	0.947	0.522	1.717
	Type of transplantation: double LTx	−0.719	0.362	3.939	1	0.047	0.487	0.240	0.991
	Sex: female	−0.249	0.352	0.501	1	0.479	0.780	0.391	1.553
	Age at baseline (years)	0.003	0.014	0.053	1	0.817	1.003	0.976	1.031
	FEV_1_ at randomization (L)	−0.128	0.285	0.202	1	0.653	0.880	0.504	1.537
Last step	Treatment: L-CsA-i	−0.021	0.296	0.005	1	0.945	0.980	0.549	1.748
	Type of transplantation: double LTx	−0.831	0.299	7.723	1	0.005	0.436	0.242	0.783

*While the upper part of the table shows the initial step that includes all specified covariates, the lower part shows the final step after removal of the covariates that did not have a statistically importance, predictive effect for lung function progression*.

*FEV1, forced expiratory volume in 1 s; LTx, lung transplantation; L-CsA-i, liposomal Cyclosporine A for inhalation*.

In a *post-hoc* Cox regression analysis on all-cause mortality that included underlying diagnosis as a predictor, neither the underlying diagnosis main effect, nor the interaction effect between underlying diagnosis and type of transplantation were significant.

Out of a total of 48 confirmed deaths, 39 (81%) were BOS-related and the remaining 9 (SLTX 4 of 40 and DLTX 5 of 90 subjects) were attributable to other causes. In SLTX recipients, “other”-cause deaths occurred at 7 (acute pulmonary edema), 46 (native lung adenocarcinoma), 75 (lung neoplasm), and 79 (lung tumor) months after baseline. In SLTX recipients, “other”-cause deaths were reported at 10 (anastomosal bleeding), 13 (post-transplant lymphoproliferative disorder), 18 (encephalomyelitis), 92 (cerebral hemorrhage), and 108 (terminal renal insufficiency) months after baseline. Separate Cox regression analyses were also performed for BOS-related mortality and for non-BOS-related mortality. In both analyses none of the predefined covariates had a significant predictive effect.

Median survival estimated by Kaplan-Meier survival analysis was 95 months (SLTX: 69 months; DLTX: 101 months) for BOS-related death and 118 months (SLTX: 91 months; DLTX: 120 months) for non-BOS-related death.

## Discussion

While on average lung function was lost over time, individual lung function trajectories after transplantation were highly variable. A high baseline FEV1 and male sex were associated with a larger subsequent decline of lung function. There was a highly significant association of FEV1_baseline_% with mortality risk. Each 1% loss of FEV1_baseline_% was associated with a 3.4% increased risk for mortality. SLTX recipients or patients receiving placebo compared to L-CsA-i were at risk for a significant loss of lung function defined as FEV1_baseline_ ≥20%. Finally, being a SLTX recipient was the only risk factor for mortality.

The core study 12011.201 that aimed to assess prophylaxis of BOS by L-CsA-i to improve long-term outcome included only patients who had not yet developed an allograft dysfunction. The study population thus appears to be unbiased for investigating the association between post-transplant FEV1 and mortality risk. Previous studies have focused on FEV1 decline after CLAD onset, which has been shown to be associated with outcome. Further progression of CLAD after its onset has been linked to unfavorable prognosis (5–8). The impact of FEV1 course in lung transplant recipients without allograft dysfunction has not been quantified.

However, in the contrast to patients with established CLAD, where FEV1 stabilizes or further decreases over time, the variability of lung function trajectories of patients in the early phase after transplantation poses challenges. While in some patients lung function tends to improve over a longer post-transplant period, others achieve the peak in FEV1 early after transplantation with subsequent stabilization or early decline of lung function. However, despite the heterogeneity of trajectories in the current analysis a joint model investigating the association between FEV1_baseline_% and mortality over a follow-up period of up to 10.5 years found a highly significant predictive effect (*p* < 0.001), with a predicted increase in relative mortality risk by 3.4% for every percent of the FEV1 baseline value lost, and a minimum increase by 1.6% according to the lower limit of the associated 95% CI. FEV1 decline could thus explain an appreciable proportion of the increase in risk of mortality and therefore appears to be relevant predictor of an unfavorable clinical outcome. Neither type of treatment applied in the core study (L-CsA-i or placebo) nor type of LTx (single or double) could be identified as important covariates in the event process of the joint model, also indicating that neither of them relevantly modified the association between FEV1 decline and increase in mortality risk. The use of FEV1 as a surrogate endpoint in investigations regarding the effects of an intervention administered to improve long-term outcome seems feasible.

Development of CLAD often takes several years, and thus a study investigating the efficacy of any measure preventing or delaying the onset of CLAD may be challenging to perform. In our analysis with a follow-up of up to 10 years, approximately one third developed a persistent loss of FEV1_baseline_% ≥ 20. This is likely an underestimation of the CLAD development, but it indicates the need for long study periods and high number of patients when effects of early interventions on CLAD development are assessed. Furthermore, according to the currently used criteria a significant amount of lung function has to be lost before the diagnosis CLAD can be established. Understanding the impact of FEV1 decline might have a role in patient care and might enable an early intervention that could modify the inevitable damage of respiratory function and subsequent CLAD.

Treatment (L-CsA-i or placebo) and type of LTx were identified as significant predictors of progression to a loss of FEV1_baseline_% ≥ 20, with more favorable prognoses for patients receiving L-CsA-i and/or DLTX. Furthermore, DLTX was again associated with a more favorable prognosis with respect to mortality, but treatment (L-CsA-i or placebo) had no statistically important effect. It was not the aim of the study to find further supporting data for the use of L-CsA-i in LTx recipients. The premature termination of the original study which resulted in the abortion of the investigational treatment of some of the study participants makes a long-term effect even more difficult to assess. However, the beneficial effect of L-CsA-i on development of a progressive phenotype, without seeing an effect on mortality may suggest that treatment over the full study period of 52 weeks or beyond, or a higher number of participants may have had beneficial effects on mortality.

A high baseline FEV1 was associated with a subsequent FEV1 decline in our analysis. The study lacks an explanation for this association. However, it seems likely that in patients with high FEV1 there is more absolute lung volume, which can be lost until a critical level of lung function is reached. In patients with low baseline FEV1 a minor decline may already have deleterious effects. So not the high FEV1 itself might be a risk factor for subsequent decline, but the fact that there is enough room for a decline, whereas in patients with low FEV1 the magnitude of subsequent decline in the setting of graft failure is usually lower.

The results of our study should be interpreted in view of the study design and its limitations. As addressed above studies on prevention of CLAD with the inclusion of patients 6–32 weeks after transplantation poses challenges. While some have already peaked in lung function and stabilized or even started to decline already, in others lung function still increased. However, despite varying lung function trajectories, preventive measures are likely to be of success when initiated early after transplantation. In contrast to our early establishment of a baseline FEV1 the current CLAD criteria rely on the assessment of the two best measurements after transplantation as baseline FEV1. However, the best post-operative FEV1 may develop years after transplantation and can only be assessed retrospectively at a time when graft dysfunction might has started already. In this line, the use of the current CLAD criteria and starting a preventive trial at the time of one of the two best FEV1 after transplantation, respectively, would be difficult the realize.

Moreover, our analysis relies on the applied treatment (L-CsA-i vs. placebo). Therefore, changing retrospectively the baseline to a post-randomization time point in a substantial proportion of study participants would lead to a confoundeing between the applied treatment (placebo vs. L-CsA) and lung function assessments, which would have biased the personal best FEV1 value. Since FEV1 baseline was dependent on the inclusion in the study, a decrease of FEV1 ≥ 20% in our study cannot be compared to CLAD criteria and is likely an underestimation of the true CLAD incidence of our cohort.

In conclusion, due to the close association between FEV1 decline and increase in mortality risk observed in this investigation, post-LTx FEV1 may be a valid predictor of mortality and may thus be a suitable surrogate endpoint in the investigation of the effect of an intervention in the prevention of CLAD. This might enable early intervention that could modify the inevitable damage of respiratory function which is of enormous medical need. Furthermore, design of clinical trials maybe facilitated and studies less demanding for investigators and participants which might result in more prevention and treatment trials.

## Data Availability Statement

The raw data supporting the conclusions of this article will be made available by the authors, without undue reservation.

## Ethics Statement

The studies involving human participants were reviewed and approved by LMU Ethics Committee. Written informed consent for participation was not required for this study in accordance with the national legislation and the institutional requirements.

## Author Contributions

NK, VM, CK, PJ, JP, PU, AS, JM-Q, AV, JB, and CN participated in research design and performance of the research. NK, AG, KM, AV, JB, and CN participated in the writing of the paper. NK, AG, KM, VM, CK, PJ, JP, PU, AS, JM-Q, AV, JB, and CN participated in data analysis. All authors contributed to the article and approved the submitted version.

## Funding

This study was funded by Zambon S.p.A., Milan, Italy.

## Conflict of Interest

NK reports research funds and honoraria for consulting from Breath Therapeutics, a Zambon company, Italy. CN reports honoraria for consulting and advisory boards from Breath Therapeutics, a Zambon company, Italy and PARI GmbH, Germany. The remaining authors declare that the research was conducted in the absence of any commercial or financial relationships that could be construed as a potential conflict of interest.

## Publisher's Note

All claims expressed in this article are solely those of the authors and do not necessarily represent those of their affiliated organizations, or those of the publisher, the editors and the reviewers. Any product that may be evaluated in this article, or claim that may be made by its manufacturer, is not guaranteed or endorsed by the publisher.
